# Optimal use of external demands in hospitals - a Delphi study from the Netherlands

**DOI:** 10.1186/s12913-016-1315-8

**Published:** 2016-02-20

**Authors:** Louise H. K. Blume, Nico J. H. W. van Weert, Jamiu O. Busari, Diana Delnoij

**Affiliations:** Zuyderland Medisch Centrum, Postbus 5500, 6130 MB Sittard-Geleen, Netherlands; Tilburg School of Social and Behavioural Sciences, Tranzo, Scientific Center for Transformation in Care and Welfare, Tilburg University, P.O. Box 90153, 5000 LE Tilburg, Netherlands; Q! B.V., Annie Romein-Verschoorlaan 11, 6532 SN Nijmegen, Netherlands; National Health Care Institute (Zorginstituut Nederland), Postbus 320, 1110 AH Diemen, Netherlands

**Keywords:** Delphi, External demands, Priority, Hospitals, Guidelines, Risks

## Abstract

**Background:**

Regulatory authorities focus on promoting compliance of hospitals with a variety of external demands. Due to the amount of these external demands, hospitals might prioritise to cope with the external demands. In this study, we explore to what extent a risk-based prioritisation system developed by one Dutch hospital, is applicable in other hospitals as well. The specific research question was: can a risk-based prioritisation system help hospitals cope with the pressures of external demands?

**Methods:**

We conducted a Delphi study, containing three rounds with seven quality and safety managers. All participants were experienced in coping with external demands in Dutch hospitals in general and their own hospital specifically. These experts were granted access to a sample selection of a database containing about 1500 external demands (January 2014). Prior to the Delphi study, a baseline measurement was carried out, where all participants answered open-ended questions aimed at identifying existing practices, possible challenges concerning external demands and to prepare the survey for the group Delphi study.

**Results:**

We identified a high level of consensus during our Delphi research. The experts agreed that at present, Dutch hospitals do not cope with external demands systematically. The participants agreed that the database and the risk-based prioritisation system are useful tools to cope with the amount of external demands and indicated that they would also like to use these tools themselves in the future.

**Conclusions:**

In this study, the participants agreed that the database and the risk-based prioritisation system are both applicable and useful tools to cope with the amount of external demands. Further research addressing the use of the risk-based-priority system for specific subsets of external demand is also needed.

## Background

Nowadays, hospitals have to deal with many external demands. These external demands are specific requirements and expectations that healthcare institutions must adhere to in order to obtain or renew licensure to practice. A large amount of external demands are clinical guidelines, which were originally developed to synthesize scientific evidence, professional experience and patient preferences. They were meant to promote the use of new knowledge and achieve standardisation to decrease variation in the procedures [1]. Moreover, they were intended to support decision-making by professionals and patients in the doctor’s office or at the bedside. However, clinical guidelines have taken on significantly more meaning today in hospitals and other healthcare institutions.

The use of clinical guidelines, or specific aspects of those guidelines, became obligatory in many countries [2, 3]. Research around the world has been devoted to investigate the implementation of, and adherence to, guidelines in various healthcare organisations. Failure to implement guidelines has been reported in the literature for instance in the UK for fragility fracture prevention guidelines [4] and in Turkey, North America, Jordan and Tanzania [5–8]. In these studies, different causes for non-adherence were identified, such as a weak evidence base for recommendations and the lack of standardised communication pathways. A few studies specifically identified the great number of guidelines as one of the reasons for non-adherence. In a Canadian study, non-adherence to guidelines in the intensive care unit was examined. One of the conclusions was that there were too many guidelines to adhere to [9]. The same was mentioned in a study conducted in 2010 in the United States for nutrition guidelines [6]. Another Canadian study showed that there were gaps between the recommendations in several critical care nutrition guidelines and the reality at the bedside [10].

Strikingly, nearly all of these studies analysed the adherence to just one guideline or a set of guidelines around one topic. Similarly, models and theories about hospitals adherence to guidelines, such as Graham et al’s knowledge-to-action cycle [11], focused on specific topics and specific guidelines, and not on the overarching question of how to apply the total volume of clinical guidelines. In our study, we focus on the problem of hospital compliance with all applicable external demands, as this defines the regulatory burden the Governing Board has to accommodate. These include clinical guidelines but non-clinical regulations as well, such as standards, guidance, indicators, laws, rules, regulations, (volume and quality) norms from insurance companies, letters and reports from the inspectorate. In the Dutch context, all of this is mainly enforced by the Dutch Health Care Inspectorate (IGZ) [12].

Full hospital compliance with all of the mandatory external demands is a widespread problem and is not unique to the Netherlands. However, we use the Netherlands as an empirical illustration of the issue. A brief description of the healthcare system and hospitals in the Netherlands is provided below for a better understanding of the Dutch context.

### Healthcare system and hospitals in the Netherlands

Dutch general hospitals are privately owned and funded through a premium-based insurance system [13]. The quality of healthcare delivery in the Netherlands is regulated by the IGZ, which functions under the auspices of the Ministry of Health, Welfare and Sport. For its regulatory tasks, the Inspectorate promotes compliance with external demands [12, 14], by using various enforcement measures ranging from the provision of recommendations, imposing fines, up to compliance orders. The conditions under which these measures are operationalized are governed by two laws - the Quality Act and Medicines Act.

Regulation by the IGZ has intensified over the last decade for all external demands and in its wake, the compliance with external demands became mandatory in 2011. This meant that all care services had to be provided in accordance with these demands [12], resulting in new problems for hospital management. This is not only a Dutch phenomenon, as many governments around the world are consolidating the regulation of medical professionals and institutions [15]. This study took place in multi-specialty facilities. The majority of Dutch hospitals - just like in other Western countries - are multi-specialty facilities that combine acute and chronic care as well as diagnosis and treatment in an increasingly multidisciplinary environment. This phenomenon might contribute to the amount of different external demands that a hospital has to comply with. In the Netherlands, the development of guidelines is not coordinated centrally [14]. It is, therefore, difficult for hospitals to have a complete overview of all external demands.

Many institutions, medical speciality organisations, professional groups, researchers, healthcare providers, insurers and patient organisations are actively engaged in the development of guidelines for clinical practice. The state only provides a legislative framework for external demands while the details are worked out by professionals and providers [13]. If any group engaged in guideline development fails to develop a field norm or standard reference, the IGZ has the mandate to develop such a norm itself. This particular approach to healthcare system governance is based on negotiations and consensus-seeking between the state, professional bodies, healthcare providers, patients and insurers – i.e., between the state and the ‘societal partners’ in healthcare [13]. However, there is no mandated list of parties that are considered to have development authority with regard to clinical guidelines or other external demands on hospitals.

For the quality of care and hospital performance to be consistently organised in Dutch hospitals, the collaboration between the Executive Board and the medical specialists is needed and this is formally regulated in Admission Agreements [16]. However, this traditional way of collaboration is shifting. In the Dutch Quality Act of 1996, the Governing Board has been named as the legal entity responsible and accountable for the quality of care. This central role for the “Governing Board” is underpinned in the so-called Governance Code of the Trade Association of Care and the IGZ [12]. It is questioned whether the Admission Agreements provide hospital Executive Boards with sufficient legal options to assume their responsibilities regarding quality and safety [17].

### Problem statement and research questions

In a recent study, healthcare guideline developers stated that guidelines aid the decision-making process for physicians and patients [18]. However, the sheer amount of external demands threatens to render them impractical for daily use. This poses the question how objective prioritisation can take place; a question highly relevant both to both hospitals and regulators.

In order to stay up-to-date with external demands and to demonstrate to the IGZ and the outside world that the Governing Board is in control, the Zuyderland Medical Centrum (Zuyderland MC), a large teaching hospital in which two of the authors worked during the study (LHKB and NJHWW), created a database (bearing the name *l’artis)*. This database lists all external demands Dutch hospitals have to adhere to in an effort to make them structurally available within the hospital, and to facilitate prioritisation [19]. Departing from the risk-based prioritisation system that has been developed in Zuyderland MC, we aim to investigate whether other Dutch hospitals are subject to similar problems and whether the risk-based prioritisation system developed in Zuyderland MC could help them in coping with external demands, too. In this sense, this is a feasibility study analysing whether a solution developed in one hospital could be implemented in other hospitals and deliver useful results.

For the purpose of this study, we formulated the following research question:

‘Can a risk-based prioritisation system help hospitals cope with the pressures of external demands?’ In addition, we developed the following sub-questions:

1. Do the participating hospitals experience similar challenges in complying with external demands?
2. Can managers from other hospitals use the risk-based prioritisation system that Zuyderland MC developed and how useful will they find it?
3. Can they assess the external demands which were collected and disseminated by Zuyderland MC in the same way?

## Methods

In the Netherlands, there are currently more than 1500 external demands used to guide and monitor the performance of hospitals [19]. In an attempt to conform to these regulations, Zuyderland MC introduced several regulatory procedures. One of these procedures entailed the compilation of all the external demands into the l’artis database and development of a risk-based prioritisation system. In this system, the Governing Board can directly determine that an external demand has a high priority by giving the score 1000. Other external demands can also receive a risk-score from seven staff members of the quality and safety department, after which it the risk-score is discussed with the management and afterwards adopted by de Governing Board. Every risk score is based on the sum of five different risk descriptions, namely: sanctions enforced by the IGZ, risks for patients, financial risks, reputational risks and risks related to the quality of care. Each external demand receives a risk score between zero and 1000. Scores above 150 indicate serious risks in the five areas. External demands with a risk score above 150 are implemented with priority and the progress of is monitored quarterly. A protocol and scoring table exists to apply the risk-based system, but for our study, a simplified protocol was developed, since only one individual instead of a group of people, registered the scores.

This research used a Delphi study to test whether this risk-based prioritisation system is suitable for other hospitals as well. The participating hospitals were not randomly selected, as explained in step 1, and the guidelines were partially randomly selected, as explained in step 4. The following six research steps were completed during this research.

### Study population (step 1)

The eight hospitals of the Association of Tertiary Medical Teaching Hospitals (STZ) in the southern region of the Netherlands, as well as a general hospital in the region, were invited to test the risk-based prioritisation system and the database. The group consisted of one academic hospital, one small hospital, and seven non-academic teaching hospitals. The experts were quality and safety managers, responsible for handling external demands in their own hospital. Two hospitals did not participate in this study due to time constraints, therefore seven hospitals agreed to participate in this study. The response rate of the seven participants was 100 % for all six research steps.

### Research instruction protocol (step 2)

To guide participants in using the Zuyderland MC risk assessment method, an instruction protocol including six steps for the risk-based prioritisation system was developed by the author LHKB. The protocol was tested on comprehensibility, logic and language by four Zuyderland MC employees, none of whom had been previously involved in risk-based prioritisation: a secretary, a policy employee, the Quality and Safety department manager and a policy employee of the Governing Board. The protocol was adjusted and retested by the policy employee of the board.

### Baseline measurement (step 3)

To identify existing practices and possible challenges concerning external demands and to prepare the survey for the group Delphi study, a baseline measurement was carried out. The experts received seven open-ended questions by email, with the purpose of discovering if and how hospitals deal with external demands at present.

### Applying the risk-based prioritisation system (step 4)

The research instruction protocol guided participants from a broad set of external demands to those of highest priority, using the five risk descriptions, namely sanctions enforced by the IGZ, risks for patients, financial risks, reputational risks and risks related to the quality of care. The participants received a sample selection of 250 of the 1515 external demands in the *l’artis* database. To ensure that sufficient discussion would arise and to avoid that too few priorities would be left after the random selection, we decided to select all 72 external demands that had previously been assessed as a priority in the Quarterly report by Zuyderland MC. The remaining 178 were randomly selected in the database of external demands. The aim of the sample selection was to reflect the reality on a smaller scale and the task was to apply the research instruction.

Firstly, the participants entered the database and individually screened 250 external demands to select a maximum of 40 external demands for further assessment. Only the 40 demands that the participants selected for their hospital, had to be scored by them using the five risk descriptions.

### The group Delphi study (step 5)

In step five, the Delphi technique was applied; this is a commonly used method to gather information from an expert panel. The Delphi method was chosen as it facilitates the discovery of strengths and weaknesses of a new system. It helps to seek answers to improve the understanding of developments, forecast, problems, opportunities and solutions [20]. The unique feature of the original Delphi technique is the repeated questioning, whereby the interim results from earlier rounds are presented together with new questions. The face-to-face communication is replaced by distant communication and characterized by anonymity. The original procedure can easily take 2 to 9 months [21].

To reduce the length of the period needed for the study, the Delphi-members agreed to participate in a group Delphi study on the 27^th^ of January 2014. The difference in this approach compared to the original Delphi technique is that experts are physically present in the same location and that the different rounds of the Delphi study can be carried out in sequence. Therefore, the duration of the Delphi study can be reduced to a single day [21]. The literature gives no indication that the shorter duration affects the results. It does affect the anonymity, which is not given during a group Delphi study. The main communication during a group Delphi study is based on questionnaires so that tight structuring beforehand is necessary and the statements of the questionnaires need to be prepared largely in advance [21].

To test the logic, comprehension and language of the statements, five think aloud tests were carried out with non-participants of the Delphi study, after the statements for round one were developed. A think aloud test is a form of cognitive testing in which participants verbalize their thoughts as they move through the questionnaire with the aim to identify and subsequently improve the items that are perceived as confusing [22]. The feedback was processed after each test before the next person was subjected to the think aloud test. After all test were carried out, the Delphi method was applied.

### Data collection (step 6)

The participants were gathered in one location, at Zuyderland MC, but placed separately in different rooms, where three rounds of Delphi research were conducted. The SurveyMonkey’s tool was used for each round [23]. The research was carried out in Dutch. The statements of round 1 were entered beforehand while the statements of round 2 and 3 were added during the Delphi execution day. These latter statements were based on the results of round 1 and 2. The research team used the break between each session to perform an analysis, and to develop new statements for the next session. Some examples of the statements that were developed included ‘It is important that we as a hospital meet external demands’, ‘It is important that hospitals know which mandatory external demands for critical processes they have implemented in their hospital’ and ‘The risk-based prioritisation system can be useful for hospitals to manage the external demands’.

In order to reach consensus and to compare the results, participants responded to assumptions using a two-item scale (‘agree’ and ‘disagree’). A ‘no opinion’ option was not included, but text boxes for comments were provided where applicable (in about half of the statements). The responses were calculated and defined as achieving consensus (≥80 % agreement) or non-consensus (<80 % agreement). Participants rated their agreement with the statements in each round. In the case of non-consensus or numerous comments, the statements were refined for the following round [21]. Bar charts representing the distribution of responses were generated using the SurveyMonkey software. Panel response rates remained 100 % across all rounds. At the end of the day, a group discussion took place.

### Independence

Two of the authors worked in Zuyderland MC (LHKB and NJHWW) and the third one (DMJD) is both a Professor at Tilburg University and the head of the quality programme of the National Health Care Institute, a government agency. To enhance independence of this study, an advisory committee supervised this research. The developer of the risk-based scoring system (NJHWW) did not participate in the development of the Delphi questions and in the analysis of the results. He did participate in the Delphi study itself. Also, a draft article was reviewed by an IGZ advisor.

### Ethics statement

Under the Dutch law, a Delphi study in which healthcare professionals and managers participate is not subject to ethical approval. Nevertheless, prior to commencing this study, the authors checked at the Medical Ethical Committee Atrium-Orbis-Zuyd whether ethical approval was needed and it was confirmed that is was not needed for this study.

## Results

### Results baseline measurement

Participants declared that they did not have an overview of all existing external demands, especially not when it came to the clinical guidelines developed by professional associations. They indicated that they prioritised the implementation of mandatory external demands on critical processes. External demands high on the agenda of the IGZ were listed by most hospitals. The majority of participants stated that it was not clear who is responsible for the distribution and implementation of external demands within their hospital. According to them, the current arrangement was too decentralised and unknown. Participants stated that there is a need for more structure concerning the use of external demands.

### Results risk-based prioritisation system

After the application of the research instruction protocol, the seven participants logged their selections and risk scores in a spreadsheet and delivered it to the researcher. One external demand was chosen by all seven participants and it contained quality indicators for infection prevention in hospitals. It was published by the Society for Hygiene and Infection prevention in Healthcare (VHIG) and the Dutch Society for Medical Microbiology (NVMM). Three external demands were selected by six hospitals and three external demands were selected by five hospitals. The selected external demands focus on infection prevention, quality standards, Dutch standards (NEN norm) and the safety management systems. Half of the external demands, 125, were not prioritised by a single hospital.

Approximately 50 % of the selected external demands are directly related to the IGZ. One hospital selected 12 external demands related to the IGZ and another selected 25. Half of the selected external demands were applicable to the hospital as a whole, not merely to a specific department or specialism. Two external demands were labelled as top priority of the Governing Board by four hospitals. Both are external demands focused on safety management systems.

### Results Delphi

Seven experts participated during the group Delphi Study. Six of the seven experts were on the same location and one expert participated from another location. They all fully completed the three rounds of the survey individually. At the end of the day, a discussion took place on the statements of non-consensus of the third Delphi round. The participant at the other location did not participate in that discussion. Overall, the participants achieved consensus on most statements. The consensus of the three Delphi rounds is displayed in Table 1.

**Table 1:** Distribution of consensus among the statements in three Delphi rounds

Round	Statements	Consensus	No consensus	Open questions
Round 1	35	28	4	3
Round 2	36	22	11	3
Round 3	11	5	5	1

The analysis of the first round results led to a number of more specific new statements in round 2. In the second round, 19 questions were formulated to provide in-depth details for the results of round 1 and a further 17 were added. Round three focused on items that needed clarification to achieve final consensus. Eleven statements were presented, all of which were completed. In total, full consensus was obtained for the essential aspects.

#### Consensus

Participants agreed that the infrastructure for external demands in Dutch hospitals needs to be arranged more effectively. An overview is needed for compliance management, to prioritise external demands and to be proactive. They stated that it is important to monitor external demands regularly to stay informed about national developments and concurred that a central external demand officer should be instated in each hospital.

According to the participants, governing boards currently cannot know the degree of compliance within their own hospital, since it is unclear where new and existing guidelines for medical specialists are collected and how the professionals use guidelines. They agreed that one can monitor whether the everyday practice is in accordance by structuring the internal dissemination and the implementation of external demands. Participants established that the registration of the implementation status is desirable within their hospitals and that the database could facilitate this.

According to the participants, an external demand receives more attention in hospitals when enforcement measures by the regulator (IGZ) are in place. Nevertheless, they also stated that it is not always clear which external demand will be actively enforced next. Participants noted that unexpected visits by the regulator are useful in consolidating the importance of these demands. Participants estimated that it is impossible to implement everything due to the amount of external demands and they feel that more focus is necessary. They noted that the standards for judging and deciding of the IGZ should be set up thoroughly and, in their opinion, this had not always been the case in the past. The majority of the participants did not recognise the IGZ- enforced issues as the most important ones for quality and safety, and thus questioned whether implementation of these external demands was the most effective contribution to risk reduction and quality improvement in their hospital.

During the discussion it became clear that the perceived role of the IGZ is influential, however, enforcement by the IGZ provokes reactive policies from hospitals when it comes to setting priorities. Participants stated that they would like to be proactive in their management of locally foreseen risks but feel that, because of IGZ policy, they are often forced to be reactive as they are behind on compliance. Participants also chose whether or not to implement external demands and declared that it is important to capture these substantiated choices. They saw the necessity for other bodies, such as the Dutch Hospital Association, National Health Care Institute, Royal Dutch Medical Association, and the Knowledge Institute of Medical Specialists, to understand the need to choose which external demands should be implemented with priority. All of the participants claimed that the Dutch Association of Hospitals should help hospitals to communicate these choices to the IGZ.

#### Scoring risks

Consensus was reached that the database and the risk-based prioritisation system of Zuyderland MC was applicable and useful for other hospitals to manage external demands. Participants indicated that their prioritisation of external demands was influenced by those of the IGZ as well as of other enforcers, ensuring that these topics were on the list.

Participants recommended that more than one person should perform the prioritisation in order to enhance reliability and that in addition to staff employees, some physicians and other clinical experts should be involved. The five risk descriptions formed an adequate basis to prioritise the external demands based on risks. According to 100 % of the participants, a new element called ‘actuality’ should be added to get a better picture of the risk. The descriptions ‘scope’ and ‘publisher’ of external demands could also be added as to assess risks according to 86 % of the participants.

Participants also stated that nationwide agreements are needed concerning the production, the dissemination and the validation of external demands applicable within the Dutch hospital sector. Attention should be paid to the design of external demands, for example by making it mandatory to use state-of-the-art methods for clinical guideline development. Until this happens, they agreed that working on the database together was useful and also recommended regular exchange between them to discuss high priorities. They concurred that this exchange will support the choices participants make concerning external demands and it could reduce the risk of missing significant external demands.

All participants agreed that the Governing Board should be able to add priorities next to the risk-based prioritisation system and that the board should bear ultimate responsibility for ensuring compliance while the medical specialists share the responsibility for managing external demands.

#### Non-consensus

The participants could not agree on whether it was too complicated to communicate choices of implementing external demands to patients, public, insurers and other bodies. The majority of the participants believed that if properly substantiated, the IGZ, public or insurers may show an understanding if hospitals decided not to implement certain external demands. It was emphasized that this understanding would depend on the external demand and the communication strategy used.

The participants disagreed on whether the date of publication affected their prioritisation. Also, during round 1 the participants disagreed on the description ‘financial risk’ and ‘reputation risks’. During the Delphi rounds, discussion arose on the necessity to assess the external demands and whether specific background knowledge is needed. It was apparent in the final discussion that one must scan the external demand text, i.e., to the health problem it addresses, to assess the risk involved in non-adherence, but not read it in detail, as many external demands have hundreds of pages elaborating on technical and procedural solutions.

## Discussion

Our main research question was: ‘Can a risk-based prioritisation system help hospitals cope with the pressures of external demands?’. Overall, the results of this study show that Dutch hospitals do experience challenges in complying with external demands and that a risk-based prioritisation system could help them to cope with this pressure. The power of the database and the risk-based scoring system lies in the local embedding, as they provide the Governing Board with the possibility to act proactively. However, effects of other possible implementation procedures, e.g., one where medical specialists take a proactive role, were not included in this research. Further research is needed on the tension between a top-down approach by the Governing Board, and the bottom-up approach in which medical specialists tackle specific risks and challenges in their local practice.

The study shows that the participating hospitals experience great difficulties in coping with a large amount of external demands, which is in line with what Carthey et al. [24] stated for healthcare compliance in the UK. As mentioned in the introduction, guidelines were originally developed to summarize existing scientific evidence to reach standardisation [1] and to support decisions for professionals and patients in the doctor’s office and at the bedside. Some parts of guidelines are advisory and others mandatory [18]. Half of the problem of non-compliance is that guidelines are non-applicable, not known about, out of date or unworkable [25]. This seems to be neglected when guidelines and other external demands are given a mandatory status. If the expectations were defined more precisely, external demands can be addressed more efficiently and compliance could be improved.

This study also shows that the infrastructure for meeting external demands in Dutch hospitals needs to be arranged more effectively. As pointed out in the introduction, the Governing Board is named as the legal entity that is responsible and accountable for the quality of care. Even though Governing Boards and managers are aware of many external demands, it is hardly possible to know and monitor all of them. The overview, and therefore awareness, is missing [26].

The findings of this study show that the database and the risk-based scoring system are useful to deal with external demands on a local level. However, the tension between the local approach and the national approach can arise, as it is expected that Governing Boards comply instead of prioritise. The supervision of the IGZ will still take place and enhancements may follow. Whether prioritisation is desirable on a national level was not a part of this study and it was also not addressed whether priorities can better be balanced on local or national level. A national debate about these issues is desirable and is currently being initiated by the authors.

Another finding from our study was the substitution effect of enforcement. According to the participants, the external demands received more attention in hospitals if enforcement measures by the regulator were at hand. This in itself is in accordance with the aim of external enforcement [12]. Activities from IGZ and other regulators, even unexpected visits, were perceived as useful support by participants to achieve compliance, if they addressed the external demands which had priority on local level. However, in areas which were not chosen as local priority, activities of regulators urge Governing Boards to re-prioritize to the detriment of local needs. This substitution might decrease the impact of compliance management on actual quality improvement and risk containment. It would be interesting to conduct further research on the balance between the internal supervision, where the local risks are close and the external supervision, based on national level considerations.

The study shows that working on the database jointly with other hospitals could be useful and that regular exchange between hospitals is desirable to discuss high priorities and national developments. Hospitals can share the same source of information about external demands and use similar strategies for prioritising and coping with demands. At the moment, four hospitals have agreed to continue the work on the database and the risk-based prioritisation system together. The feasibility and success of implementing this system may improve by involving the target group during development and distribution. This contributes to efficiency and capacity building and might mutually facilitate improvement of risk-assessment as hospitals can compare their scores. Further research that addresses the use of the risk-based-priority system for clearly defined subsets of external demand is needed.

Internationally, this study is also interesting. However, regulators around the world need to ask themselves to what extent enforcement measures are beneficial in ensuring compliance, when does it just pull health professionals away from other, equally important tasks. Greenhalgh et al [27] stated that to “equate ‘quality’ in clinical care with strict adherence to guidelines or protocols, however, robust these rules may be, is to overlook the evidence on the more sophisticated process of advanced expertise” (p.3). Enforcement can lead to undesirable side effects, Robben states, such as strategic behaviour, manipulation and fraud [28]. For countries, where central coordination of the development of external demands is missing, the problem is probably similar to the Dutch situation. These countries could also benefit from the results of this study, as a risk-based priority system might be a possible solution for them, too. Further research is needed on this topic.

### Strengths

Some of the strengths of this study include the full participation of all responders (no drop out) through the study. Also, the Delphi questions from round 1 were prepared critically, and 15 different testers contributed their expertise to the statements. Furthermore, the participants examined a large set of external demands for the risk-based prioritisation system to make the results representative of the entire set. Finally, the findings from our study may be useful for other Dutch hospitals and for hospitals across countries, as the study did not deal with the specific content of the external demands but challenged the question from a governance perspective.

### Limitations

Before interpreting our findings, several limitations should be considered. One being, that Delphi studies generate expert consensus and therefore rank low as scientific evidence. Another limitation is that we included quality and safety managers with a positive attitude towards external demands and that one person carried out the risk-based prioritisation system per hospital. A further limitation is that the medical teaching hospitals from the south of the Netherlands work together in various fields and this might influence the strong agreement on many issues.

The existence of the database adds value to hospitals; however, it can still be improved. To ensure that experts can assess and prioritise thoroughly, it is desired to add a summary of each external demand to the database. Debating the findings of this study during a round table session with various stakeholders from in and around hospitals may be useful since these barriers should be addressed nationwide.

## Conclusions

At present, Dutch hospitals are not structurally dealing with external demands. During the Delphi study, the participants agreed that the database and the risk-based prioritisation system of Zuyderland MC are both applicable and useful tools to cope with the amount of external demands, and that they would like to use these tools in the future. At the moment, four hospitals have agreed to work on the database and the risk-based prioritisation system together.
